# The effects of sub-chronic calcium treatment on ethanol-induced dopamine elevation and the alcohol deprivation effect in the rat

**DOI:** 10.1038/s41398-026-03804-1

**Published:** 2026-01-10

**Authors:** Karin Ademar, Klara Danielsson, Bo Söderpalm, Louise Adermark, Mia Ericson

**Affiliations:** 1 Addiction Biology Unit, Department of Psychiatry and Neurochemistry, Institute of Neuroscience and Physiology, The Sahlgrenska Academy, University of Gothenburg, Gothenburg, Sweden; 2 Department of Addiction Disorders, Sahlgrenska University Hospital, Region Västra Götaland, Gothenburg, Sweden; 3 Department of Pharmacology, Institute of Neuroscience and Physiology, The Sahlgrenska Academy, University of Gothenburg, Gothenburg, Sweden

**Keywords:** Molecular neuroscience, Physiology, Addiction

## Abstract

Alcohol use disorder (AUD) is a serious mental health condition and a risk factor for morbidity and preterm death. The drug acamprosate (Campral® – calcium-bis[N-acetylhomotaurinate]) is one of few pharmacological treatments available for AUD. Recent research suggests that the properties of acamprosate may be attributed to calcium, but the acute and long-term effects by calcium supplementation on ethanol-induced dopamine release and relapse-like drinking is not fully known. We used in vivo microdialysis and the alcohol deprivation model, to further define the interaction of local or systemic calcium and ethanol on accumbal dopamine and taurine levels, and to outline the impact of acute and repeated calcium treatment on dopamine and the alcohol deprivation effect (ADE) in male Wistar rats. The role of calcium was further studied by local administration of an L-type Ca^2+^ channel (LTCC) blocker. The results demonstrate that acute local administration of calcium in naïve rats increased nucleus accumbens extracellular dopamine levels, and prevented ethanol from further increasing dopamine. In addition, the ethanol-induced elevation of taurine was delayed in animals receiving calcium. Following sub-chronic systemic calcium administration, the dopamine-elevating property of calcium was lost. The LTCC inhibitor nicardipine decreased accumbal dopamine levels and prevented both calcium and ethanol from altering dopamine output. Acute systemic calcium administration abolished the ADE in treatment-naïve rats, but not in rats pretreated with sub-chronic calcium. Taken together, the results suggest that acute properties of calcium abolish ethanol-induced effects within the mesolimbic dopamine system, while there is an indication of tolerance development to both the dopaminergic and behavioral ethanol-related effects of calcium, thus mimicking the outcomes previously observed with acamprosate.

## Introduction

Excessive, compulsive and uncontrolled alcohol drinking are characteristics of alcohol use disorder (AUD), which causes significant morbidity and mortality worldwide [1]. The development of AUD is a dynamic process ultimately leading to a chronic and relapsing brain disorder with a transformed neurophysiological state [2]. The pharmacotherapies available for treatment of AUD have limited effect sizes and to develop new effective treatments, the mechanisms of both the available treatments, as well as that of ethanol’s interaction with the brain reward system, need to be elucidated further.

Ethanol interacts with several neuronal circuits including the mesolimbic dopamine system, with dopaminergic projections from the ventral tegmental area (VTA) to, primarily, the nucleus accumbens (nAc), leading to increased extracellular dopamine levels within the nAc [2, 3]. By means of in vivo microdialysis, ethanol has also been demonstrated to increase extracellular taurine levels in the nAc, which is a partial glycine receptor (GlyR) agonist [4, 5]. The ethanol-mediated dopamine elevation is proposed to involve activation of accumbal GlyRs, as administration of the GlyR antagonist strychnine blocks the ethanol-induced dopamine increase [6]. Interestingly, the alcohol relapse-preventing drug acamprosate (calcium-bis(*N*-acetylhomotaurinate); Campral®) appears to partly mimic ethanol within the mesolimbic dopamine system. There is evidence that acamprosate increases both dopamine and taurine levels in the nAc, the former by involving accumbal GlyRs and nicotinic acetylcholine receptors (nAChRs) in the VTA [7–11]. Furthermore, beyond the early proposals of acamprosate acting to reduce the ethanol-induced hyperexcitability emerging during withdrawal [12–14], the mechanistic principles of the drug have been attributed to the calcium component of the substance [15–17]. Thus, acamprosate’s mechanisms of action still remain unknown [18, 19].

Calcium, administrated both systemically and locally within the nAc, has been found to produce acamprosate-like effects in both neurochemical and behavioral animal models. In a recent mechanistic study, we found that calcium increases extracellular dopamine levels in the nAc in a similar GlyR sensitive manner as previously observed with acamprosate [8, 10]. In addition, acamprosate and calcium treatment produce similar results in various animal models examining ethanol-intake and –seeking [11, 15] and ethanol-induced cognitive deficits [16]. In the alcohol deprivation effect (ADE) model, which assesses relapse-like or uncontrolled drinking behavior, calcium treatment has however generated conflicting outcomes [11, 15, 17], and in animal models examining the effects of acamprosate on ethanol intake and relapse-like drinking, the animals often develop tolerance following long-term treatment [9, 20, 21].

**Fig. 1:**
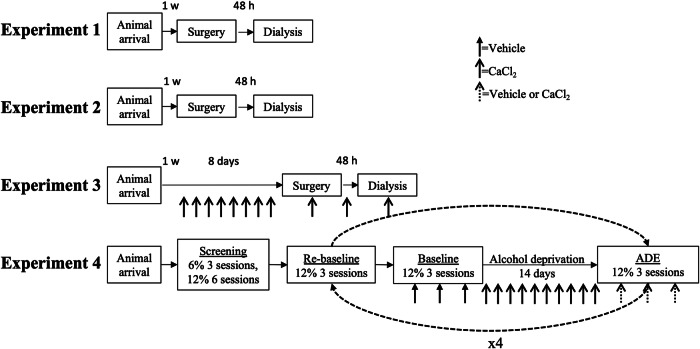
Schematic overview of experimental design. Outline of the different experiments performed in the study. For all the experiments (1–4), animals were allowed to adapt to the new environment for at least one week (1 w) prior to initiation of any experiments. For the microdialysis experiments (1–3), animals were allowed to recover for 48 h (48 h) following the dialysis probe implantation surgery before the dialysis experiment started. For the alcohol deprivation experiment (4), animals were facing repeated alcohol deprivation periods to establish robust relapse-like drinking behavior. Arrows indicate vehicle or drug injections as depicted by the different arrow styles. ADE alcohol deprivation effect.

In the present study, we aimed to investigate the effects of calcium on ethanol-induced dopamine and taurine output in the nAc following acute local administration in naïve male rats and in male rats pre-treated with an L-type Ca^2+^ channel (LTCC) blocker. Additionally, we wanted to define if calcium acutely affects the alcohol deprivation effect (ADE), and if repeated systemic calcium administration affects its ability to increase accumbal dopamine or its effect on the ADE. Thus, we further aimed to elucidate the effects of calcium in the context of the pharmacological effect of acamprosate. To this end, four separate experiments were conducted using in vivo microdialysis and behavioral studies as further outlined in Fig. 1. First (experiment 1), in vivo microdialysis was employed to define acute effects by local nAc calcium treatment on baseline and ethanol-induced dopamine and taurine levels in the nAc. Second (experiment 2), also using in vivo microdialysis, the role of LTCCs in calcium- and ethanol-induced dopamine release was explored. Next, the role of repeated systemic intraperitoneal (i.p.) calcium treatment on baseline dopamine levels in the nAc was defined using in vivo microdialysis (experiment 3). Lastly (experiment 4), the acute and repeated exposure to calcium on relapse-like ethanol intake was assessed. We hypothesized that acute calcium administration to naïve rats increases nAc dopamine levels, prevents ethanol from further increasing dopamine and blocks the ADE. We further hypothesized that rats repeatedly treated with calcium develop tolerance to all these effects.

## Materials and methods

### Animals

Male Wistar rats (Inotiv, Horst, the Netherlands) were used for all experiments. In experiment 1–3, 115 animals at age 9–10 weeks, weighing 270–380 g were group-housed from arrival until microdialysis probe placement surgery at constant room temperature (20–22 °C) and humidity (55–65%) at regular 12 h light-dark conditions (lights on at 7:00 AM and lights off at 7:00 PM). In experiment 4, 36 animals, age 6–7 weeks and weighing 150–170 g at the initiation of the study, were single-housed (cage size: 55$*$35$*$20 cm) at constant room temperature (20–22 °C) and humidity (50–55%) at reversed 12 h light-dark conditions (lights on at 10:00 PM and lights off at 10:00 AM). All rats included in the different experiments were allowed at least one week of acclimatization to the animal facility, while being group-housed (cage size: 55$*$35$*$20 cm), before any experiments were initiated. All animals were randomly allocated to the experimental groups in each experimental series. The researcher performing the experiment was not blinded to drug administration, but analysis of the data was performed by a blinded researcher. Throughout the experiments, animals had *ad libitum* access to standard rodent chow and water. The experiments in the study were approved by the Ethics Committee for Animal Experiments in Gothenburg, Sweden, and conducted in accordance with the relevant guidelines. The study is reported in accordance with ARRIVE guidelines.

### Drugs and solutions

Ethanol (96%; Kemetyl AB, Haninge, Sweden or Kiilto Clean AB, Täby, Sweden) was diluted to 300 mM in Ringer’s solution (consisting of (in mM): 140 NaCl, 1.2 CaCl_2_, 3.0 KCl, 1.0 MgCl_2_) and perfused via the microdialysis probe (nAc) or diluted in tap water to 6 or 12% (v/v) and added to the home cage fluid bottles. The use of 300 mM ethanol is approximately equivalent to 2.3 g/kg i.p., yielding 60–65 mM in the tissue surrounding the dialysis probe [22]. CaCl_2_$*$2H_2_O 0.5 mM (Fisher Scientific, Gothenburg, Sweden) was dissolved in Ringer’s solution and perfused via the microdialysis probe (nAc) or dissolved in 0.9% NaCl when administered systemically in a concentration of 73.5 mg/kg (2 ml/kg, i.p.). The dose of 73.5 mg/kg calcium contains equivalent amounts of Ca^2+^ ions as 200 mg/kg acamprosate (0.499 mmol/kg). Nicardipine (Sigma-Aldrich, Stockholm, Sweden) was dissolved in pre-warmed Ringer’s solution, diluted to 100 µM and perfused via the microdialysis probe (nAc).

### In vivo microdialysis

Rats were surgically equipped with a custom-made dialysis probe two days prior to the in vivo microdialysis experiment. Rats were deeply anesthetized with 4% isoflurane (Baxter, Kista, Sweden) during induction before being mounted onto a stereotaxic instrument (David Kopf Instruments, AgnTho’s, Ldingö, Sweden) and placed on a heating pad for sustained body temperature. The skull was exposed through a sagittal incision and a total of three holes were drilled, one for the probe placement and the additional ones for anchoring screws. An I-shaped probe with a semi-permeable membrane, with an active space of 2 mm and a molecular cut-off of 20 kDa, was gently lowered into coordinates approximating the nAc core-shell borderline region (A/P: + 1.85 mm, M/L: −1.4 mm relative to bregma, D/V: −7.8 mm relative to dura [23]). The probe and the anchoring screws were fixed to the skull using Harvard cement (DAB Dental AB, Gothenburg, Sweden). Rats were single-housed (cage size: 40$*$24$*$18 cm) and allowed to recover for 48 h prior to the experiment. To provide analgesia during and after surgery, and to avoid dehydration, rats received systemic administration of Metacam® (1 mg/kg, 2 ml/kg, Boehringer Ingelheim, Ingelheim/Rhein, Germany), and local application of Marcain® (Aspen Pharma, Dublin, Ireland) alongside the surgical incision, together with 2 ml 0.9% NaCl s.c. in the early phase of the surgical procedure.

On the experimental day, the inlet and outlet tubes of the probe were connected to a microperfusion pump (U-864 Syringe Pump, AgnTho’s, Lidingö, Sweden) via a swivel allowing the rat to move around freely in its home cage. Each experimental day included 5–6 rats, and group allocation was randomized. All treatments were included on each experimental day whenever feasible. Prior to sampling, the probes were perfused with a Ringer’s solution at a rate of 2 µl/min for 2 h to obtain balance in the fluid exchange over the dialysis membrane. The equilibrium phase was followed by baseline sampling, and acute pharmacological treatment was administered when a stable dopamine baseline ( ± 10%) was achieved. Basal dopamine levels did not differ between groups (average 2.11 ± 0.21 nM). Dialysate samples (40 µl) were collected every 20 min throughout the experiment. After the experiment, animals were sacrificed and brains were removed and placed in fixative (Accustain, Sigma-Aldrich, Sweden). Probe placement was verified using a vibroslicer (Campden Instruments Ltd, Leicester, UK) 4–7 days later and rats with misplaced probes or substantial hemorrhage were excluded. A total of 9 animals were excluded from the microdialysis study due to incorrect probe position or bleeding.

Experiment 1 – *effects of acute administration of CaCl*_*2*_ *and ethanol on dopamine and taurine levels in the nAc in naïve rats*: CaCl_2_ (0.5 mM), ethanol (300 mM), CaCl_2_ + ethanol (CaCl_2_ alone for 40 min after which ethanol and CaCl_2_ were co-perfused) or Ringer’s solution were locally perfused in the nAc of naïve rats, and extracellular dopamine and taurine levels were evaluated. The expected maximum of ethanol-induced dopamine elevation occurs 40 min after start of the perfusion [5, 24], while the expected maximum of ethanol-induced taurine elevation occurs 20 min after start of the perfusion [4, 5, 25]. Thus, a time-point comparison between treatments were performed 80 min after start of the experiment for dopamine levels, and 60 min after start of the experiment for taurine levels in the nAc. The time interval for calcium pre-treatment before the start of ethanol perfusion was based on that the expected maximum of calcium-induced dopamine elevation occurs 40–60 min after start of the perfusion [10].

Experiment 2 – *effects of acute administration of nicardipine and CaCl*_*2*_ *or ethanol on dopamine levels in the nAc in naïve rats*: Nicardipine (100 µM), nicardipine + CaCl_2_ (nicardipine alone for 80 min after which CaCl_2_ (0.5 mM) and nicardipine were co-perfused), nicardipine + ethanol (nicardipine alone for 80 min after which ethanol (300 mM) and nicardipine were co-perfused) or Ringer’s solution were locally perfused in the nAc in naïve rats. Extracellular dopamine levels were evaluated. The time interval for nicardipine pre-treatment before the start of calcium or ethanol perfusion, was based on that a robust effect of the nicardipine-induced dopamine reduction was warranted.

Experiment 3 – *effects of acute administration of CaCl*_*2*_ *and ethanol on dopamine levels in the nAc in rats administered with CaCl*_*2*_ *or saline for ten days*: For 10 days, naïve rats received CaCl_2_ (73.5 mg/kg, 2 ml/kg i.p.) or vehicle (0.9% NaCl, 2 ml/kg i.p.). On the 9^th^ day of injections rats went through surgery and were equipped with a microdialysis probe, and on the 11^th^ day extracellular dopamine levels were measured by means of in vivo microdialysis in the nAc. On the experimental day, all rats received an injection of CaCl_2_ (73.5 mg/kg, 2 ml/kg i.p.).

### Biochemical assays

The samples were analysed using high-performance liquid chromatography (HPLC). The levels of dopamine were detected and quantified online using an electrochemical detector and an external standard containing 3.25 nM of dopamine [26]. The levels of taurine were detected and quantified at a later stage, using a fluorescent detector and two external standards containing 500 or 1000 nM of taurine [27]. Thus, the dialysate samples were split for separate analysis of dopamine and taurine, and sodium azide (50% v/v) was added to the split sample for amino acid preservation. The amino acid sample was frozen (−20 °C) until analysis. All chromatograms were analyzed using Thermo Scientific Chromeleon Chromatography Data System (CDS) software (CHROMELEON7).

### Long-term alcohol consumption with repeated deprivation periods

The alcohol deprivation effect (ADE) was evaluated following repeated alcohol deprivation phases. Rats were placed in a two-bottle-choice intermittent ethanol consumption paradigm with access to ethanol for three 24 h sessions per week (bottles added on Monday, Wednesday and Friday at the beginning of the dark period (10 a.m.)). Rats were habituated to an ethanol solution containing 6% (v/v) for one week, after which they received a 12% (v/v) solution for the rest of the experiment. Following a total of four weeks with ethanol access, rats were exposed to one week of baseline injections (vehicle; 0.9% NaCl) prior to the first alcohol deprivation period of two weeks. After the first deprivation period, rats were put on the intermittent ethanol intake paradigm again for another two weeks before the next deprivation period. The paradigm was repeated and the effect of chronic and acute treatment with calcium was evaluated at the end of the fourth deprivation period. Rats were evenly divided into three treatment groups based on their ethanol intake during baseline (n = 12 rats/group). The sub-chronic treatment with calcium was initiated on the fifth day of the two-week alcohol deprivation period. Water intake was monitored on daily basis and body weight on weekly basis. Rats were exposed to ethanol over a period of 23 weeks, including the deprivation phases.

### Statistics

Data were analyzed using GraphPad Prism, version 10 for Windows (GraphPad Software, Inc., San Diego, CA, USA). Baseline dopamine values were calculated for each animal using an average of time-points −40, −20 and 0, after which all individual values were related to this average. Two-way analysis of variance (ANOVA) with repeated measures (treatment group*time) over the relevant period of time, and followed by Tukey’s post hoc test when applicable, was used for statistical analysis of microdialysis data in experiment 1–3. One-way ANOVA followed by Tukey’s post hoc test was used for analysis of microdialysis data at the relevant time-point in experiment 1. Unpaired and paired *t*-tests were used for statistical evaluation of the microdialysis data in experiment 3 and ADE in experiment 3. Effect sizes for dopamine (time-point 80) microdialysis were reported as Hedges’ g for independent group comparisons. Ninety-five percent confidence intervals were derived from noncentral-t/F distributions. All data, but the effect sizes, are expressed as means±standard error of the mean (SEM) and statistical significance considered a probability value (*p*) less than 0.05. Sample size was not predetermined by statistical methods but based on prior experimental experience (7–12 rats for in vivo microdialysis and 12 for ADE behavior).

## Results

### Acute calcium administration abolishes ethanol-mediated dopamine, but not taurine, elevation in the nAc of naïve rats

**Fig. 2:**
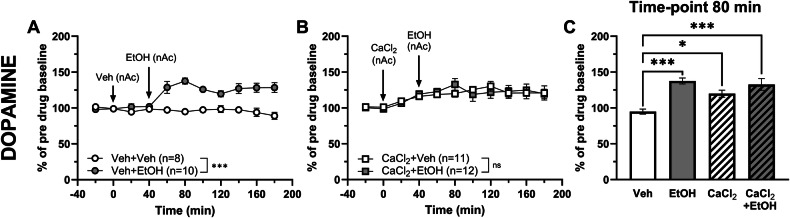
Ethanol-induced dopamine increase in the nAc is prevented by acute calcium treatment in naïve rats. Time-course graphs of extracellular dopamine output in the nAc using reversed microdialysis after administration of (**A**) vehicle (Ringer’s solution) alone or in combination with ethanol (300 mM), and (**B**) CaCl_2_ (0.5 mM) alone or in combination with ethanol. Ethanol significantly increased accumbal dopamine levels compared to vehicle treated rats (**A**), while no significant difference in dopamine levels was observed between rats receiving CaCl_2_ alone compared with rats receiving the addition of ethanol (**B**). Bar graph of (**C**) accumbal dopamine levels at time-point 80 min (40 min following the addition of ethanol) demonstrated that CaCl_2_ prevents ethanol from further elevating nAc dopamine in naïve rats. Arrows indicate time-point for drug administration. * denotes a significant effect compared to vehicle. All data are presented as mean values ± SEM. *p < 0.05, ***p < 0.001. CaCl_2_ calcium chloride, EtOH ethanol, nAc nucleus accumbens, Veh vehicle.

Since acamprosate has been found to block the ethanol-evoked dopamine increase in nAc [9], we aimed to examine the effects of calcium on ethanol-induced dopamine elevation. Consistent with our previous studies, both ethanol (300 mM) [24, 28] and CaCl_2_ (0.5 mM) [10] elevated accumbal dopamine levels when administered locally in the nAc (two-way ANOVA with repeated measures_t = 0–180 min_; EtOH: treatment effect F_(1, 16)_ = 37.16 *p* < 0.001, time effect F_(3.60, 57.6)_ = 5.05 *p* = 0.002, interaction F_(10, 160)_ = 8.04 *p* < 0.001; Fig. 2A; CaCl_2_: treatment effect F_(1, 17)_ = 25.16 *p* < 0.001, time effect F_(2.70, 45.9)_ = 1.73 *p* = 0.178, interaction F_(10, 170)_ = 2.76 *p* = 0.004; data not shown in graph). Further, perfusion of CaCl_2_ prior to ethanol blocked the ethanol-induced dopamine elevation (two-way ANOVA with repeated measures_t = 0–180 min_: treatment effect F_(1, 21)_ = 0.003 *p* = 0.957, time effect F_(2.90, 70.0)_ = 7.82 *p* < 0.001, interaction F_(10, 210)_ = 0.733 *p* = 0.693; Fig. 2B). When assessing peak dopamine elevation (t = 80 min), dopamine was significantly increased in all treatment groups compared to vehicle treatment (one-way ANOVA_t = 80 min_: F_(3, 37)_ = 8.64 *p* < 0.001; Tukey’s post hoc test: vehicle vs EtOH *p* < 0.001, vehicle vs CaCl_2_ *p* = 0.041, vehicle vs CaCl_2_ + EtOH *p* < 0.001; Fig. 2C), but there was no significant difference between treatment groups (Tukey’s post hoc test: CaCl_2_ vs CaCl_2_ + EtOH *p* = 0.395; EtOH vs CaCl_2_ + EtOH *p* = 0.935; Fig. 2C). When estimating the effect sizes, vehicle vs EtOH revealed the largest effect Hedges’ g = 3.44, 95% CI [2.01, −4.88], vehicle vs CaCl₂ Hedges’ g = 1.69, 95% CI [0.66, 2.71], CaCl₂ vs CaCl₂ + EtOH, Hedges’ g = 0.53, CI [−0.28, 1.33] and CaCl₂ + EtOH vs EtOH, Hedges’ g = 0.21, CI [−0.60, 1.01].

**Fig. 3:**
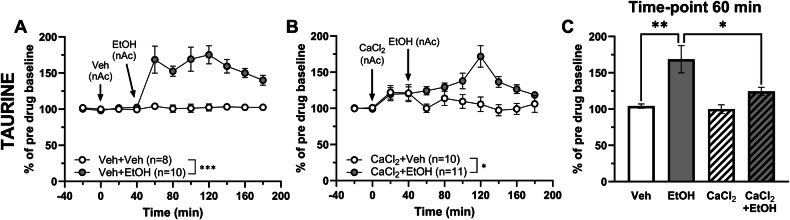
Ethanol-induced taurine increase in the nAc is not prevented by acute calcium treatment in naïve rats. Time-course graphs of extracellular taurine levels in the nAc using reversed microdialysis following administration of (**A**) vehicle (Ringer’s solution) alone or in combination with ethanol (300 mM), and (**B**) CaCl_2_ (0.5 mM) alone or in combination with ethanol. Ethanol significantly increased the accumbal taurine levels compared to vehicle treated rats (**A**), and a significant difference in taurine levels was also observed between rats receiving CaCl_2_ alone compared with rats receiving the addition of ethanol (**B**). Bar graph of (**C**) accumbal taurine levels at time-point 60 min (20 min following the addition of ethanol) demonstrated a significant delay in the onset of ethanol-induced taurine elevation in the nAc in the presence of CaCl_2_. Arrows indicate time-points for drug administration. * denotes a significant effect by treatment. All data are presented as mean ± SEM. *p < 0.05, ***p < 0.001. CaCl_2_ calcium chloride, EtOH ethanol, nAc nucleus accumbens, Veh vehicle.

As both acamprosate and the combination of CaCl_2_ and *N*-acetylhomotaurine have taurine-elevating properties [10], the nAc taurine levels of the same batch of rats were evaluated. Local perfusion of 300 mM ethanol significantly increased extracellular taurine levels in the nAc compared to vehicle-treatment (two-way ANOVA with repeated measures_t = 0–180 min_: treatment effect F_(1, 16)_ = 53.04 *p* < 0.001, time effect F_(2.96, 47.3)_ = 8.86 *p* < 0.001, interaction F_(10, 160)_ = 7.59 *p* < 0.001; Fig. 3A). Local perfusion of CaCl_2_ produced a transient increase in accumbal taurine over the first two time-points, which did not sustain throughout the recording (two-way ANOVA with repeated measures_t = 0–180 min_: treatment effect F_(1, 14)_ = 1.08 *p* = 0.316, time effect F_(3.21, 44.9)_ = 1.07 *p* = 0.374, interaction F_(10, 140)_ = 1.59 *p* = 0.116; data not shown in graph). Pre-treatment with CaCl_2_ did not prevent ethanol from elevating the accumbal taurine levels (two-way ANOVA with repeated measures_t = 0–180 min_: treatment effect F_(1, 19)_ = 6.24 *p* = 0.022, time effect F_(3.43, 65.1)_ = 5.56 *p* = 0.001, interaction F_(10, 190)_ = 5.21 *p* < 0.001; Fig. 3B). However, the onset of the ethanol-induced taurine output was delayed in CaCl_2_ pre-treated animals as compared to naïve rats receiving ethanol, and the relative increase in taurine was significantly lower when assessed at expected peak amplitude (t = 60 min) (one-way ANOVA_t = 60 min_: F_(3, 35)_ = 8.69 *p* < 0.001; Tukey’s post hoc test: vehicle vs EtOH *p* = 0.001, EtOH vs CaCl_2_ + EtOH *p* = 0.021; Fig. 3A-C).

### Calcium channel antagonism using nicardipine prevents accumbal calcium- and ethanol-evoked dopamine release

**Fig. 4:**
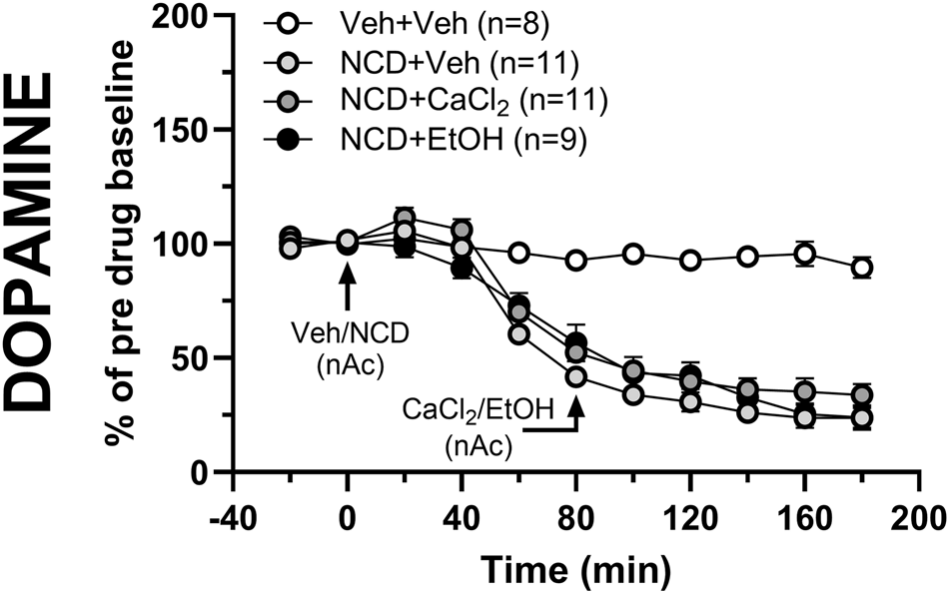
Accumbal pre-treatment with nicardipine prevents both calcium- and ethanol-induced dopamine elevations in the nAc of naïve rats. Time-course graphs of extracellular dopamine output in the nAc using reversed microdialysis following administration of vehicle (Ringer’s solution), nicardipine (100 µM) alone or in combination with CaCl_2_ (0.5 mM) or ethanol (300 mM). No significant differences in dopamine levels were observed between rats receiving nicardipine alone compared with rats receiving the addition of CaCl_2_ or ethanol. Arrows indicate time-point for drug administration. All data are presented as mean ± SEM. CaCl_2_ calcium chloride, EtOH ethanol, nAc nucleus accumbens, NCD nicardipine, Veh vehicle.

Based on the experiments performed, calcium appears to play a key role in regulating ethanol-induced dopamine release. To further outline the mechanisms underlying CaCl_2_-induced dopamine increase rats were pre-treated with the dihydropyridine and L-type Ca^2+^ channel (LTCC) blocker nicardipine (100 µM). Perfusion of nicardipine robustly reduced the extracellular dopamine level in the nAc compared to vehicle-treated controls (two-way ANOVA with repeated measures_t = 0–180 min_: treatment effect F_(3, 35)_ = 44.21 *p* < 0.001, time effect F_(3.80, 132.9)_ = 204.4 *p* < 0.001, interaction F_(30, 350)_ = 16.09 *p* < 0.001; Fig. 4). Co-administration of CaCl_2_ (0.5 mM) was not sufficient to elevate dopamine compared to nicardipine-treatment alone. Furthermore, pre-treatment with nicardipine followed by ethanol (300 mM) co-perfusion did not increase dopamine compared to control (two-way ANOVA with repeated measures_t = 80–180 min_: treatment effect F_(2, 28)_ = 2.63 *p* = 0.090, time effect F_(3.50, 97.91)_ = 243.5 *p* < 0.001, interaction F_(20, 280)_ = 1.54 *p* = 0.066; Fig. 4). Thus, LTCCs appear to play a key role in regulating both basal dopamine levels, and the ethanol-induced elevation of dopamine.

### Calcium-induced dopamine elevation is abolished in rats treated with calcium for ten days

**Fig. 5:**
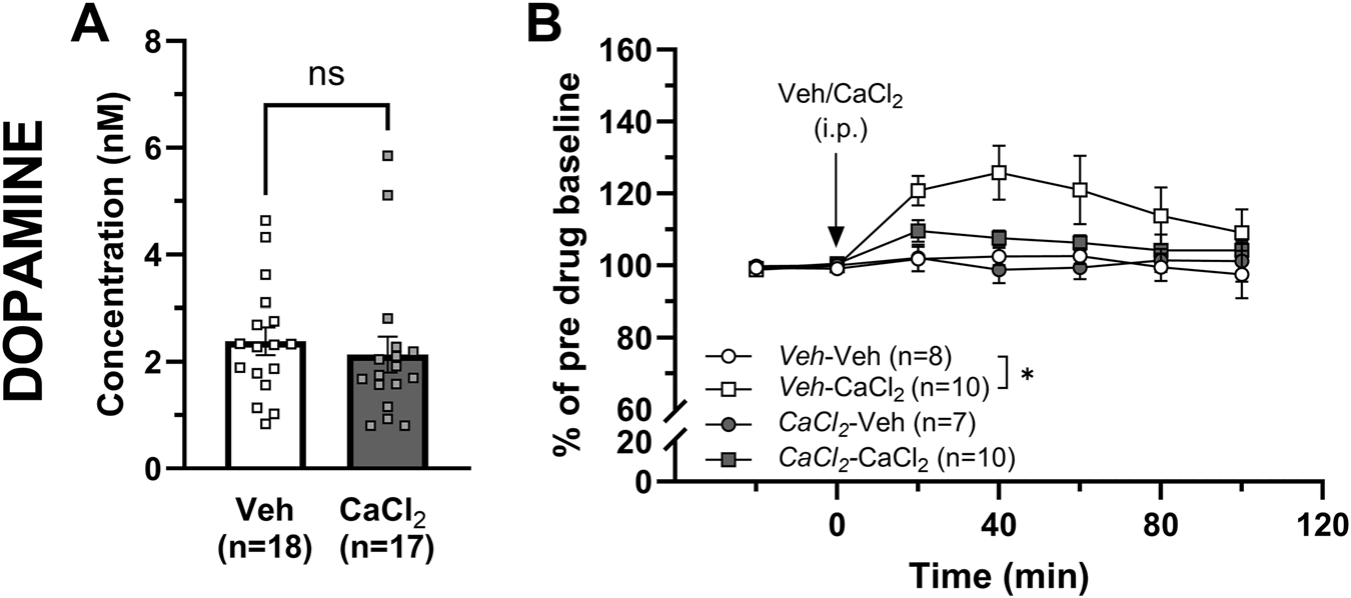
An acute calcium injection does not evoke dopamine elevation in the nAc of rats repeatedly treated with calcium for ten days. **A** Basal extracellular levels of dopamine at time-point 0 presented as concentration in nM in the nAc of all rats pre-treated with either CaCl_2_ (73.5 mg/kg) or vehicle (0.9% NaCl) for ten days. **B** Time-course graphs of extracellular dopamine levels in the nAc in rats receiving an acute systemic i.p. injection of vehicle (0.9% NaCl) or CaCl_2_ (73.5 mg/kg). CaCl_2_ produced a significant dopamine increase in rats pre-treated with vehicle, but not in rats pre-treated with CaCl_2_ for 10 days. Arrow indicates time-point for drug administration. Legend name represents which pre- and acute treatment each group received. * denotes a significant result compared to vehicle. All data are presented as mean ± SEM. *p < 0.05. CaCl_2_ calcium chloride, i.p. intraperitoneal, nAc nucleus accumbens, ns non-significant, Veh vehicle.

In the next set of experiments, we wanted to investigate the impact of repeated systemic calcium administration on accumbal dopamine levels and whether the dopamine output observed in these rats would be altered following acute systemic calcium administration. Following ten days of pre-treatment with CaCl_2_, the microdialysate concentration of dopamine was not significantly altered (unpaired *t*-test: t_32_ = 0.587 *p* = 0.561; Fig. 5A). When challenged with a systemic injection of either vehicle (0.9% NaCl) or CaCl_2_ (73.5 mg/kg, i.p.), CaCl_2_ selectively increased dopamine in vehicle-treated rats (two-way ANOVA with repeated measures_t = 0–100 min_: treatment effect F_(3, 31)_ = 4.30 *p* = 0.012, time effect F_(6, 186)_ = 4.67 *p* < 0.001, interaction F_(18, 186)_ = 1.84 *p* = 0.023; Tukey’s post hoc test: *Veh*-Veh vs *Veh*-CaCl_2_ *p* = 0.021; *CaCl*_*2*_-Veh vs *CaCl*_*2*_-CaCl_2_ *p* = 758; Fig. 5B).

### Acute calcium administration prevents the alcohol deprivation effect while repeated administration does not

**Fig. 6:**
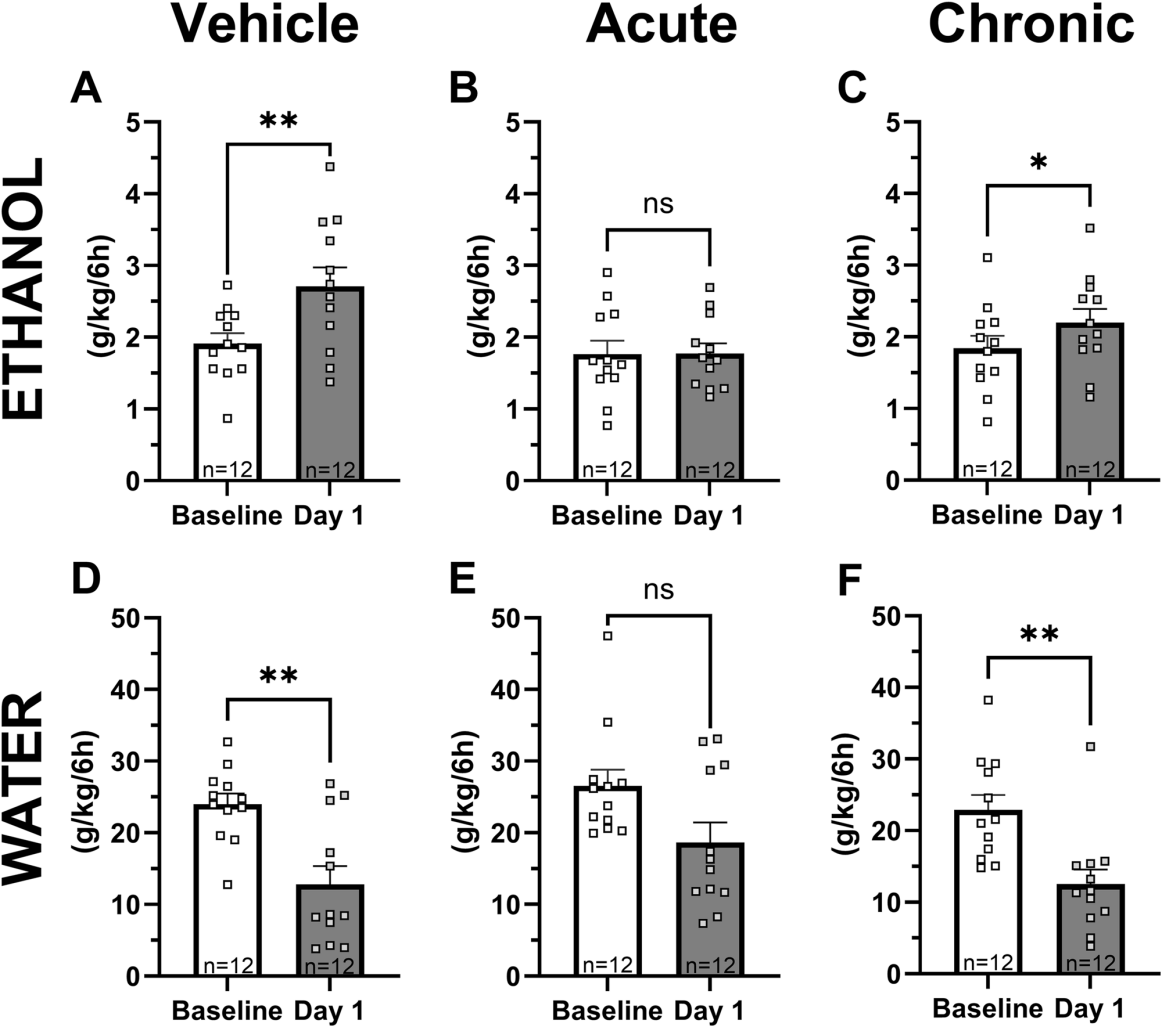
The alcohol deprivation effect is abolished by acute calcium administration, but not following sub-chronic treatment. Mean intake of ethanol and water (g/kg/6 h) of the last three sessions of the baseline period compared with the first drinking session following the two weeks of ethanol deprivation (**A-F**). A significant increase in ethanol intake was seen in vehicle (0.9% NaCl) treated rats (**A**), and in rats repeatedly treated with CaCl_2_ for ten days (**C**). However, acute CaCl_2_ treatment prevented the ADE (**B**). Water intake was reduced in vehicle treated rats (**D**) and in rats treated with CaCl_2_ for ten days (**F**), while no difference in water intake was found in rats acutely treated with CaCl_2_ (**E**). *p = 0.05, **p < 0.01. ADE alcohol deprivation effect, CaCl_2_ calcium chloride.

Since calcium treatment in the ADE model has shown both a positive outcome [15, 17] and an absence of effect [11], both acute and repeated calcium administration were assessed using the model with repeated deprivation phases. When reintroducing ethanol after a two-week long abstinence period, a significant increase in ethanol consumption was found in the vehicle treated rats as compared to their baseline ethanol intake (paired *t*-test: t_(11)_ = 3.44, *p* = 0.006; Fig. 6A). In rats treated acutely with CaCl_2_ prior to the test, no increased ethanol consumption was observed (paired *t*-test: t_(11)_ = 0.033, *p* = 0.975; Fig. 6B) indicating an abolished ADE. However, ethanol intake was significantly increased in the rats that received CaCl_2_ treatment for ten days before testing for the ADE (paired *t*-test: t_(11)_ = 2.51, *p* = 0.029; Fig. 6C). Simultaneously, water intake decreased in both the vehicle treated rats (paired *t*-test: t_(11)_ = 4.22, *p* = 0.001; Fig. 6D) and in rats repeatedly treated with CaCl_2_ (paired *t*-test: t_(11)_ = 3.83, *p* = 0.003; Fig. 6F) compared to baseline intake, while no significant difference was demonstrated in rats receiving acute treatment with CaCl_2_ (paired *t*-test: t_(11)_ = 2.14, *p* = 0.056; Fig. 6E). Thus, these findings suggest that acute calcium administration prevents the ADE, whereas this is not the case with repeated calcium treatment.

## Discussion

The mechanistic principles of acamprosate have during recent years been ascribed to the calcium part of the drug [15–17]. The overall aim of this study was to further outline the role of calcium in relation to ethanol-mediated dopamine elevation as well as relapse-like drinking, following both acute and repeated administration in the rat. The data presented here show that acute calcium treatment not only elevates extracellular dopamine levels in the nAc and prevents ethanol from further elevating the accumbal dopamine levels, but also abolishes the ADE. In addition, acute calcium treatment did also alter the taurine-elevating property of ethanol in the nAc. Moreover, blockade of LTCCs prevented dopamine signalling following both calcium- and ethanol administration. Interestingly, the effects seen with acute calcium treatment were lost after repeated administration. Indeed, following ten days of repeated systemic calcium exposure the accumbal dopamine response to acute calcium injection was significantly blunted and an ADE was present, indicating tolerance development to these effects of calcium. Overall, our data suggest that dopaminergic neurotransmission is tightly regulated by calcium and that even a minor addition affects the pharmacological effects of ethanol on accumbal dopamine and taurine levels. However, following sub-chronic treatment with systemic calcium, the dopamine-elevating property and the ability to prevent the ADE are lost.

Acute local administration of calcium in the nAc of naïve animals increased extracellular dopamine levels, in line with previous results [10]. Furthermore, when co-perfused with ethanol, ethanol was unable to further increase dopamine output. This was also highlighted when calculating effect sizes where the combination treatment compared to ethanol or calcium alone showed the smallest effect sizes, suggesting that the effect on extracellular dopamine may be saturated, occluding any additional influence of combined treatment. These observations mimic the effects previously seen following acute acamprosate administration followed by concomitant ethanol perfusion [9]. This finding thus supports the idea that calcium may play a key role in acamprosate’s mechanism of action. Although only males were used in the present study, we believe the results to be referable also to female rats since male and female rats display a similar dopamine response to ethanol [22], however future studies will need to verify this hypothesis. The increased dopamine levels following both local perfusion with calcium [10] and acamprosate [8, 10] treatment have previously been linked to GlyRs, directly or indirectly by the involvement of taurine, as pre-treatment with the GlyR antagonist strychnine blocks both effects. Further, the GlyRs have also been suggested as a key component for the increased dopamine output following ethanol exposure [4, 5, 29]. Consequently, the accumbal GlyR is a common denominator, which may explain the absence of a dopamine response after ethanol application when either calcium or acamprosate already is on board. Interestingly, the increase in nAc dopamine levels upon calcium treatment is rather modest as compared to the effect seen following acamprosate perfusion when comparing the whole treatment period [10]. This has previously been linked to acamprosate being composed of both a calcium moiety and a homotaurine molecule, and indicates that homotaurine is not inert [10]. However, the small increase in accumbal dopamine produced by calcium still has the capacity to prevent ethanol from further elevating the dopamine levels within the same brain region, indicating that activation of nAc GlyRs is central for both the calcium- and ethanol-induced dopamine output in the nAc [6, 10].

Extracellular taurine levels are known to be increased in the nAc after both local [4, 5, 25] and systemic [25, 30] ethanol administration, an effect demonstrated to be necessary for ethanol to produce a dopamine elevation [4]. Here, we investigated the accumbal taurine output following acute local calcium administration and found the extracellular taurine levels not to be elevated, which is in line with previous observations following different routes of administration [10, 11]. However, when ethanol was concomitantly perfused with calcium, an increase in the nAc taurine levels was demonstrated compared to both vehicle and calcium alone. Notably, there was a delay in the maximum peak concentration of taurine when comparing the calcium pre-treated rats and the naïve rats receiving ethanol. We have previously recognized the appearance of a biphasic shaped increase of taurine following ethanol administration, typically with a maximum of the ethanol-induced taurine elevation occurring 20–40 min after start of the perfusion [4, 25, 31], a phenomenon also observed in the present study (Fig. 3A). We can thus speculate that ethanol-induced taurine release involves separates mechanisms of actions, out of which the first phase is impacted by calcium. Indirectly these findings also indicate that it is the first phase of the taurine elevation that sparks the dopamine increase following ethanol. However, the underlying reasons for calcium’s ability to attenuate the initial peak of ethanol-induced taurine elevation can only be speculated upon and is a matter for future studies.

LTCCs play an important role in ethanol-related behaviors and dopamine release in the mesolimbic system [32]. Blockade of LTCCs using antagonists like nifedipine has been shown to reduce ethanol consumption [33, 34] and prevent the locomotor stimulatory effects of ethanol in rodents [35]. The data presented here demonstrated that inhibition of LTCCs with nicardipine significantly suppresses extracellular dopamine levels in the nAc. This finding aligns with previous research showing that mesolimbic dopamine neuron activity in the VTA to nAc pathway is regulated by calcium influx through LTCCs, specifically the Ca_V_1.2 and Ca_V_1.3 subtypes [36]. Furthermore, nicardipine administration prevented both calcium- and ethanol-induced dopamine elevation in the nAc, the latter in line with previous observations [35]. The mechanism by which LTCC blockade prevents calcium- and ethanol-mediated dopamine responses remains to be delineated. One possible explanation involves the attenuation of cholinergic receptor activation in the VTA by LTCC blockade, as it has been found that activation of cholinergic receptors by carbachol on dopaminergic cell bodies in the VTA is attenuated by LTCC blockade [37]. This may interrupt the nAc-VTA-nAc neuronal circuitry that controls ethanol-induced dopamine elevation in the nAc [38]. In the absence of nicardipine, ethanol activates this circuitry, leading to stimulation of nAChRs in the VTA and increased extracellular dopamine levels in the nAc. In the presence of nicardipine, ethanol’s ability to activate this circuitry and stimulate nAChRs in the VTA may be impaired, leading to reduced dopamine release in the nAc. This may possibly occur through reduced calcium influx through LTCCs in the nAc. Recent research has shown that dopamine release supported by functional LTCCs is regulated by various endogenous factors, which depend on both sex and the brain region studied [39]. However, Brimblecombe, Connor-Robson [39] also declared that the exact mechanisms by which LTCCs support dopamine release require further investigation.

Repeated systemic administration of calcium for ten days, before the examination of accumbal dopamine levels using in vivo microdialysis, resulted in a blunted response to an acute calcium challenge compared to calcium-naïve animals. This suggests that tolerance has developed to the calcium-induced dopamine-elevating effect after repeated treatment, which is in line with our hypothesis. In the vehicle-treated rats, an intact calcium-induced increase in nAc dopamine levels was observed, which is in line with the first experiment presented in this study using acute local administration. Interestingly, tolerance development is a phenomenon also observed in rats repeatedly treated with acamprosate [9, 21], which has been linked to acamprosate losing its ethanol-intake reducing effect [9, 21]. Theoretically, calcium may thus be the component contributing to the development of tolerance to acamprosate. Notably, differences between the rats in the vehicle and calcium pre-treated groups could not be related to differences in basal extracellular levels of dopamine.

Alcohol use disorder is a multidimensional human mental disorder, where the clinical picture and the progression of the disease differ among the affected individuals. Based on its complexity, it is thus impossible to model all aspects of the disorder in animals. However, a well-used model for relapse-like drinking in the human situation is the alcohol deprivation model. The alcohol deprivation effect (ADE) is a phenomenon where a temporary increase in voluntary ethanol intake is observed following a period of deprivation, and has a high face and predictive value for the clinical setting [40, 41]. Here, we evaluated the ADE after either acute or repeated calcium treatment and found that calcium administrated to treatment-naïve rats abolished the ADE, while rats previously exposed to calcium injections presented an ADE. The altered ADE in the calcium exposed rats may be related to the attenuated calcium-induced dopamine release observed following the long-term systemic calcium treatment using in vivo microdialysis as discussed above. Thereby, there is an indication of development of tolerance to the calcium-induced effects in the mesolimbic dopamine system, and the rewarding effects of ethanol are most likely no longer reduced [42]. In support of this idea are studies demonstrating tolerance development to both the ethanol intake reducing effect of acamprosate and no clear suppression of the ADE following longer periods of treatment [9, 20], as well as an altered ADE following repeated calcium administration before the period of alcohol deprivation started [11]. In addition, acute or shorter periods of treatment with calcium (15; the present study) or acamprosate [11, 15, 43, 44] are effective in abolishing the ADE. Hence, the loss of efficacy over time using calcium or acamprosate is proposed to be attributed to calcium. To be noted, long-term alcohol consumption with repeated deprivation phases is considered to resemble the clinical situation most closely [40], which was the approach used here. When we previously assessed calcium on the ADE after repeated drug treatment and using only a single deprivation phase [11], we observed the same results as in this study, i.e. no suppression of the ADE. This suggests that, although rats facing repeated deprivation periods are recognised with a more pronounced addictive profile with compulsive drinking despite harmful consequences and loss of typical circadian drinking pattern [44, 45], repeated calcium treatment is ineffective in preventing relapse-like drinking and has only an acute effect.

In conclusion, the alcohol-related effects of calcium here studied appear to be limited to acute effects in drug naïve rats, as repeated treatment show attenuated responses to calcium with regards both to accumbal dopamine elevation and to the ADE, suggesting development of tolerance. We argue that these effects may be connected to adaptations to calcium-induced effects in the mesolimbic dopamine system and should be further explored using fiber photometry. Accordingly, the outcome in the alcohol deprivation model seems to be related to dopamine mechanisms, which previously has been confirmed for acamprosate [9] and proposed for the combined administration of varenicline and bupropion [46]. Moreover, as LTCCs seem to be an interesting target for several neuropsychiatric disorders, the significance of LTCCs in regulating dopamine transmission in the mesolimbic dopamine system needs to be further investigated and validated in both ethanol consumption studies and in the evaluation of the ADE in both male and female subjects to open for its usage in a clinical setting for AUD.

## Data Availability

The data generated during the current study are available from the corresponding author upon reasonable request.
